# Evaluation of Endoscopic Findings in Gastrointestinal Tract Wall Thickening Detected on Abdominal Radiological Imaging: A Two-Center Retrospective Descriptive Study

**DOI:** 10.3390/medicina61091699

**Published:** 2025-09-18

**Authors:** Mustafa Ergin, Fatih Kıvrakoğlu

**Affiliations:** 1 Department of Gastroenterology, Aksaray Training and Research Hospital, Aksaray University, 68100 Aksaray, Türkiye; 2 Department of Gastroenterology, Osmaniye State Hospital, 80100 Osmaniye, Türkiye; drfatih112@gmail.com

**Keywords:** gastrointestinal diseases, gastrointestinal wall thickening, radiology, endoscopy, neoplasms

## Abstract

*Background and Objectives*: The clinical significance of gastrointestinal (GI) tract wall thickening incidentally detected on abdominal imaging remains unclear. This study aimed to examine the relationship between GI tract wall thickening seen in imaging and what is found during endoscopy, as well as to explore how hemoglobin, C-reactive protein (CRP), and albumin levels can help predict the presence of malignancy. *Materials and Methods*: In this retrospectively designed study, 209 patients were included who were found to have GI tract wall thickening on radiological imaging and underwent endoscopy within 90 days. Endoscopic findings and laboratory data were recorded. Patients were compared based on the presence or absence of malignancy, and a receiver operating characteristic analysis was performed. *Results*: Malignancy was detected in 8.5% and 10.9% of the upper and lower GI tract cases, respectively. In patients with upper GI tract malignancy, hemoglobin levels were significantly lower and CRP levels were higher (*p* < 0.001 and *p* = 0.015, respectively). Similarly, in lower GI tract malignancy, hemoglobin levels were lower (*p* = 0.033), whereas CRP did not show a significant difference (*p* = 0.115). Cut-off values were determined as 11.8 g/dL for hemoglobin and 40.75 g/L for albumin, and both were found to have high negative predictive values. *Conclusions*: GI tract wall thickening detected radiologically is clinically significant and should be further investigated endoscopically. Certain biochemical parameters may aid in ruling out malignancy; however, endoscopy remains essential for definitive diagnosis.

## 1. Introduction

The increasing use of abdominal radiological imaging has led to the frequent detection of incidental findings that are not directly related to the original clinical indication. Among these, gastrointestinal (GI) tract wall thickening is one of the most common abnormalities. While such findings may enable the early detection and potentially curative treatment of malignancies that could otherwise present with poor prognosis if diagnosed late [1,2], their clinical significance often remains uncertain and poses challenges for clinical management.

In the literature, various radiological threshold values for wall thickness have been reported for the esophagus, stomach, duodenum, and colon [3,4,5]. Exceeding these limits may be associated with a broad spectrum of conditions, ranging from infections and inflammatory diseases to neoplasia. However, the endoscopic and histopathological correlations of these radiological findings remain insufficiently clarified [6]. Consequently, there is no standardized approach available to guide clinicians in the management of these patients.

Even though multimodal imaging techniques like computed tomography (CT), magnetic resonance imaging (MRI), and ultrasonography (USG) provide a clear view of wall thickness, it is often important to use endoscopy to confirm the medical importance of what the imaging shows. Studies have shown that when wall thickening is found, endoscopy can uncover different health issues like malignancies, inflammation, and benign lesions [7]. So, it is very important to connect the wall thickening seen in scans with what is found during endoscopy and tissue analysis to ensure the right diagnosis and treatment for patients. Similarly, the literature suggests that we should not disregard incidental findings of wall thickening, as it could lead to the missed diagnosis of significant conditions like malignancy. In this context, if wall thickening is detected in both the lower and upper GI tracts, it is recommended to perform an endoscopic evaluation based on the nature of the radiological finding and the patient’s clinical condition [8,9]. However, there is a lack of sufficient studies on this subject, and no guidelines or recommendations exist to guide the clinical approach.

Despite frequent detection of GI tract wall thickening on abdominal imaging, prior studies have been heterogeneous and have rarely assessed upper and lower segments together while examining whether routine laboratory markers can inform malignancy risk. We therefore hypothesized that radiologically detected wall thickening would be associated with a substantial prevalence of clinically relevant endoscopic pathology, including neoplasia. In addition, we anticipated that baseline hemoglobin, C-reactive protein (CRP), and albumin would demonstrate discriminative ability primarily to exclude malignancy rather than serve as standalone screening tools. The primary aim of this study was to correlate radiologic thickening with endoscopic findings across GI segments, while the secondary aim was to assess the diagnostic performance of these laboratory parameters.

## 2. Materials and Methods

### 2.1. Study Design

This study was designed as a two-center, retrospective, and descriptive observational study aiming to analyze the outcomes of endoscopic evaluations performed in patients with radiologically detected GI tract wall thickening and to assess its contribution to the diagnosis of clinically significant lesions such as malignancy. The data for the study were retrospectively obtained from the records of patients who were referred to the gastroenterology clinics of Aksaray University Training and Research Hospital and Osmaniye State Hospital between February 2022 and April 2025 due to incidentally detected GI tract wall thickening on radiological imaging, and who subsequently underwent endoscopic evaluation. Biopsy samples were obtained from all suspicious lesions using standard endoscopic biopsy protocols, with at least 2–3 samples taken from each lesion. A total of 209 patients were enrolled, including 131 from Aksaray University Training and Research Hospital and 78 from Osmaniye State Hospital. In total, 234 wall thickening sites were identified, as some patients had more than one affected GI segment. The study was conducted in accordance with the principles of the Declaration of Helsinki following the approval of the relevant ethics committee.

To be included in the study, participants had to be at least 18 years old, have thickened walls in the upper or lower GI tract seen on imaging tests (like CT, MRI, or USG), and have had an endoscopy—either an esophagogastroduodenoscopy or an ileocolonoscopy—within 90 days after the imaging test. Wall thickening was defined according to commonly cited thresholds in the radiology literature: ≥5 mm for the esophagus, stomach, and colon, and ≥3 mm for the duodenum. These values have frequently been reported as the upper limits of normal in abdominal imaging studies [3,4,5].

**Figure 1:**
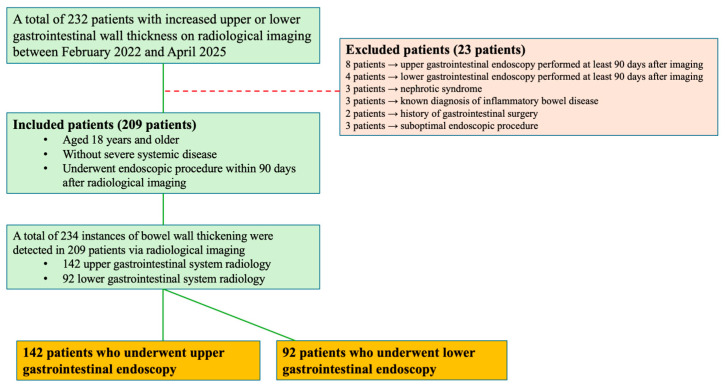
Flowchart of the study.

For the cases in the study (Figure 1); demographic data, comorbid conditions (diabetes mellitus, hypertension, cardiovascular diseases, respiratory system diseases, neuropsychiatric disorders, non-GI cancers, and other systemic illnesses), radiological imaging findings, endoscopic findings, laboratory values (hemoglobin, CRP, and albumin levels), and the interval between imaging and endoscopy were recorded. Endoscopic procedures were performed under sedation by two experienced gastroenterologists, and no procedures were conducted by residents, and the results from biopsies taken from suspicious areas were looked at later.

Excluded from the study were people who had an endoscopic evaluation more than 90 days after the radiological finding, those with systemic diseases (like congestive heart failure or nephrotic syndrome) that could cause secondary bowel wall thickening, cases with insufficient bowel distension or low-quality endoscopic images, patients with a known history of inflammatory bowel disease or previously diagnosed GI tract cancer, and individuals who had GI tract surgery before (Figure 1).

### 2.2. Statistical Analysis

Data analysis was performed using SPSS version 29.0. A priori sample size calculation was performed using *G*Power version 3.1.9.7. Assuming a medium effect size (d = 0.5), a two-tailed α of 0.05, and a power (1–β) of 0.80, the minimum required sample size was calculated as *n* = 128. Since our study included 209 patients, the sample size was sufficient to provide statistically reliable results. Descriptive statistics for qualitative data were presented as number (*n*) and percentage (%). For quantitative data, mean and standard deviation were provided for normally distributed variables, while median and minimum–maximum values were reported for non-normally distributed variables. The normality of distribution was assessed using the Shapiro–Wilk test. In comparisons of continuous variables between two independent groups, the Mann–Whitney U test was used when at least one group did not show a normal distribution. The Type I error margin (alpha) was set at 0.05. The diagnostic performance of hemoglobin, CRP, and albumin levels in predicting malignancy was assessed using Receiver Operating Characteristic (ROC) curves and the area under the curve (AUC). Sensitivity and false positive rate (1–specificity) values from the ROC curve outputs were used to determine cutoff points via the Youden Index. Sensitivity, specificity, positive predictive value, and negative predictive value were then calculated according to these cutoff values.

## 3. Results

In total, 234 thickened sites were identified among 209 patients, as some patients had involvement in more than one anatomical region. The median age of the 209 patients included in the study was 59 years (range: 18–86), with 55% (*n* = 115) being female and 45% (*n* = 94) male. Regarding body mass index (BMI) distribution, 4.3% of the patients were classified as underweight (BMI < 18.5), 33.5% as normal weight (BMI 18.5–24.9), 45% as overweight (BMI 25–29.9), and 17.2% as obese (BMI > 30). At least one comorbidity was present in 69.9% of the patients. The most common accompanying condition was hypertension (40.2%), followed by diabetes mellitus (20.6%) and cardiovascular diseases (17.2%) (Table 1).

A total of 234 instances of wall thickening were identified on radiological imaging. Of these, 60.7% were located in the upper GI tract, with CT (31.6%) and USG (28.6%) being the most commonly used imaging modalities. Wall thickening in the lower GI tract was found in 39.3% of the cases, with 29.9% checked using CT, 8.5% using USG, and 0.9% using MRI (Table 1).

Based on radiological findings, 142 patients underwent esophagogastroduodenoscopy and 92 patients underwent ileocolonoscopy. The most common finding in esophagogastroduodenoscopy was gastritis/erosion (62.7%). Other observed lesions included reflux esophagitis (18.3%), peptic ulcer (6.3%), and polyps (6.3%). The rate of malignancy detection was 8.5%, with the corpus being the most frequently affected site (3.5%) (Table 2).

In ileocolonoscopic examinations, normal findings were observed in 35.9% of the cases. Colorectal polyps were detected in 21.7%, diverticula in 12%, and malignancy in 10.9% of the cases. Among patients diagnosed with malignancy, the rectum was the most frequently affected site (5.4%). Additionally, inflammatory causes such as inflammatory bowel disease (IBD) (9.8%), non-IBD colitis (7.6%), and terminal ileitis (4.3%) were also identified (Table 3).

A total of 51 patients were found to have either malignancies or polypoid lesions, which included both neoplastic (adenomatous) and non-neoplastic types. Among esophagogastroduodenoscopies, malignancy was detected in 12 patients (8.5%) and gastric polyps in 9 patients (6.3%). Ten patients (10.9%) had lower GI tract malignancies, and twenty patients (21.7%) had colorectal polyps (Table 4). Overall, 38 of these lesions were classified as neoplastic, whereas 13 were non-neoplastic.

In patients with upper GI tract malignancy, the median hemoglobin level was found to be 10.8 g/dL, compared to 13.1 g/dL in those without malignancy (*p* < 0.001). Similarly, CRP levels were significantly higher in the group with malignancy (22.5 mg/L vs. 4 mg/L; *p* = 0.015). In patients with lower GI tract malignancy, hemoglobin levels were also significantly lower (11 g/dL vs. 13.6 g/dL; *p* = 0.033), although the difference in CRP was not statistically significant (*p* = 0.115). While the mean age was higher in patients with malignancy in both upper and lower GI tract groups, these differences did not reach statistical significance (*p* = 0.413 and *p* = 0.081, respectively). Albumin levels were significantly lower in the malignancy group for both upper and lower GI tract malignancies (*p* = 0.005 and *p* = 0.015, respectively) (Table 5).

**Figure 2:**
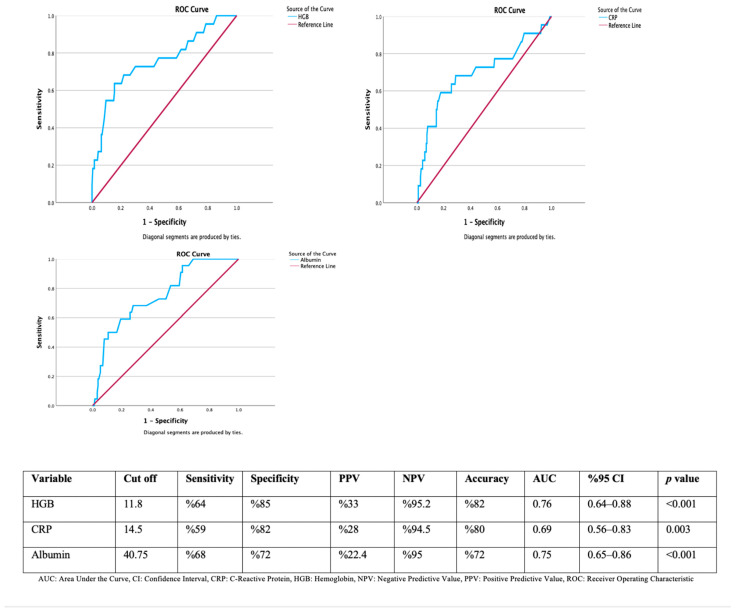
ROC curves and diagnostic performance metrics of hemoglobin, CRP, and albumin levels in predicting gastrointestinal malignancy.

In the ROC analysis, we looked at how well hemoglobin, CRP, and albumin levels can predict GI tract malignancies. The determined cut-off value for hemoglobin was 11.8 g/dL, which showed significant predictive value with 64% sensitivity and 85% specificity. The positive predictive value (PPV) was 33%, while the negative predictive value (NPV) was high at 95.2%. The overall accuracy rate was 82%, and the AUC was 0.76 (95% CI: 0.64–0.88; *p* < 0.001). For CRP, the determined cut-off value was 14.5 mg/L, yielding 59% sensitivity, 82% specificity, 28% PPV, and 94.5% NPV. The AUC was calculated as 0.69 (95% CI: 0.56–0.83; *p* = 0.003). The cut-off value for albumin was set at 40.75 g/L, which resulted in 68% sensitivity, 72% specificity, 22.4% PPV, and 95% NPV. The AUC was found to be 0.75 (95% CI: 0.65–0.86; *p* < 0.001) (Figure 2).

## 4. Discussion

This study aimed to compare radiological findings with endoscopic evaluation in patients diagnosed with GI tract wall thickening on abdominal radiological imaging. The findings obtained demonstrate that a significant proportion of patients with wall thickening had pathological lesions on endoscopy and that endoscopic examination made a substantial contribution to the diagnostic process.

GI tract wall thickening detected by radiological methods is often incidental and may not have clear clinical significance. However, in our study, most patients with thickened walls in both the upper and lower GI tracts were found to have serious pathologies like gastritis, ulcers, polyps, inflammation, or malignancies (abnormal results in 97.2% of upper GI tract and 64.1% of lower GI tract). This study demonstrates the necessity of endoscopic investigation for such radiological findings. Various studies have evaluated ileocolonoscopic findings in patients with CT-reported wall thickening. Rockey et al. reported a 67% likelihood of detecting abnormalities [10]. Moraitis et al. reported a positive correlation rate of 72%, while Wolff et al. stated that 73.9% of patients had abnormalities detected on ileocolonoscopy [11,12]. Our findings are consistent with the values reported in the literature.

In our study, lesions such as gastritis/erosion, reflux esophagitis, and peptic ulcer were frequently observed in the upper GI tract; additionally, malignancy was detected in 8.5% of the cases, and when gastric polyps are included, neoplastic and non-neoplastic findings were observed in 14.7%. Although limited, there are studies in the literature investigating the correlation between gastric wall thickening and malignancy. In one study, endoscopic procedures performed in cases with antral wall thickening detected on CT were evaluated, and it was found that wall thickening was more prominent in malignant cases compared to benign ones [13]. In another study, malignancy was detected at a rate of 29% in the upper GI tract in procedures performed due to wall thickening observed on CT [14]. Despite the high rates observed in that study, the low patient count presents a disadvantage. Similarly to our findings, another study with a comparable design found the rate of upper GI tract malignancy to be 8% [1]. Furthermore, there is also a study reporting a malignancy rate as high as 50.8% [15].

As seen, various studies have reported highly variable rates of malignancy in GI tract wall thickening detected radiologically. It is considered that several fundamental reasons underlie these differences. Firstly, the demographic characteristics of the study populations may significantly influence the results. Since the risk of malignancy increases, particularly in older age groups, studies including predominantly elderly individuals may report higher rates. In addition, studies involving symptomatic patients (e.g., those with abdominal pain, weight loss, or anemia) tend to show higher malignancy rates, while those evaluating asymptomatic patients with incidental radiological findings may report lower rates. However, it has been shown in the literature that even in asymptomatic patients, the malignancy detection rate may vary between 6% and 80% in different studies [16]. This suggests that a clinician should not hesitate to perform an endoscopy when they detect wall thickening, even in the absence of clinical symptoms. Moreover, the outcomes may also be influenced by the type of medical center conducting the study and the sample size. In tertiary referral centers, more complicated and high-risk patient groups for malignancy are encountered more frequently, while secondary care centers tend to evaluate a more general population. In our study as well, endoscopic procedures performed due to incidental radiological findings were evaluated. Additionally, the threshold value adopted for defining wall thickening may influence study outcomes. Previous radiological studies have generally considered wall thickness to be pathological when ≥5 mm for the colon, ≥5 mm for the esophagus, ≥5 mm for the distended stomach, and ≥3 mm for the duodenum [3,4,5]. Our findings are consistent with these thresholds; however, we emphasize that they are not standardized in universally accepted guidelines, which may contribute to variability across studies. Another methodological aspect is the interval between imaging and endoscopic evaluation. In our study, this period was allowed up to 90 days due to real-life logistical factors, although the median interval was considerably shorter (24.5 days for upper GI and 32.5 days for lower GI). While longer waiting times may have permitted clinical changes, there is no universally accepted guideline specifying the optimal timeframe for endoscopic evaluation after radiologically detected wall thickening. Future prospective studies should aim to clarify the most appropriate interval, likely within 30–60 days, to minimize potential bias. In a study conducted in Türkiye by Durmaz et al., the rate of esophageal cancer was found to be very high [17]. In this study, 144 patients with gastroesophageal junction and esophageal wall thickening were examined, and esophageal cancer was detected in 53 patients (37%). Furthermore, the study measured wall thickness via CT, reporting asymmetric thickening above 13.5 mm as significant for malignancy. Similarly, Tongdee et al. suggested that a CT-detected wall thickness greater than 10 mm in the gastric antrum could be used to distinguish between malignant and benign conditions [18]. In contrast, our study did not determine a specific threshold value for significant wall thickening; any GI tract wall thickening detected by imaging was considered pathological, and we assumed that endoscopic evaluation should be performed. Some studies have classified wall thickening as mild, moderate, and severe, compared pathological findings by wall thickness, and emphasized the need for endoscopy even in cases of mild wall thickening since pathological findings were observed in all cases [19].

Lower GI tract findings show a similar trend. In our study, only 15.8% of patients who underwent ileocolonoscopy were found to have normal findings, while the remaining patients had lesions such as colorectal polyps, diverticula, IBD, non-IBD colitis, or malignancy. Malignancy was detected in 10% of patients, mostly in the rectosigmoid region, and colorectal polyps were found in 21.7%. These findings support, in agreement with similar studies in the literature, that colonic wall thickening is often a finding that should not be overlooked.

In the study by Uzzaman et al., malignancy was found at a rate of 21.82% in ileocolonoscopies performed on patients with bowel wall thickening detected on CT [20]. In another study, patients with colon wall thickening detected on CT were examined, and colonoscopy revealed a malignancy rate of 28.8% and a colorectal polyp rate of 22.7%. Another study, which focused on procedures performed due to increased wall thickness on CT, found a 33% malignancy rate in the lower GI tract [14]. In the study by Akbaş et al., five-year colon wall thickening data were retrospectively reviewed, and the malignancy rate was found to be 44.8% [21]. In their retrospective study, Daniel et al. evaluated increased wall thickness throughout the entire GI tract and determined a malignancy rate of 51.6% [22]. In a prospective study conducted in India, where tuberculous peritonitis is common, 62.12% of the wall thickening was attributed to tuberculosis and 15.16% to tumors [19]. In another prospective study from India, Kumar et al. reported that among patients with ileum or cecum wall thickening detected on abdominal CT, 48% had tuberculosis, 20% had Crohn’s disease, and only one patient was found to have adenocarcinoma [23]. In the study by Wolff et al., 107 patients were looked at after the fact; those who had bowel wall thickening seen on CT and had an endoscopy within 30 days (either as an emergency or planned) were included. Wolff et al. reported that 28% of the patients had no pathology, 8% had malignancy, and 10% had IBD. The most common diagnoses were ischemic colitis (36.4%) and infectious colitis (14.9%) [12]. This led to the conclusion that urgent cases are more likely to have ischemic and infectious conditions.

The differences in rates between studies on lower GI tract wall thickening are mainly due to differences in methods used, the variety of patient groups, and the absence of consistent practices in imaging and endoscopy. This suggests that, rather than adopting a one-size-fits-all approach for every patient with detected wall thickening, an individualized assessment should be conducted, and the decision to perform endoscopy should be based on the level of clinical suspicion. Furthermore, we need to design future multicenter studies with standardized diagnostic protocols and the inclusion of appropriate control groups with normal gastric and intestinal wall morphology, which would allow direct comparison and enhance the generalizability of findings.

ROC analysis indicates that hemoglobin, CRP, and albumin levels have diagnostic value in differentiating between malignant and non-malignant conditions. The cut-off value determined for hemoglobin was 11.8 g/dL, which showed a sensitivity of 64% and a specificity of 85%, with an AUC value of 0.76 (*p* < 0.001). This indicates that hemoglobin is a good discriminative test in predicting malignancy. Similarly, a CRP cut-off value of 14.5 mg/L achieved diagnostic significance with 59% sensitivity and 82% specificity (AUC: 0.69, *p* = 0.003). Although the PPV for this parameter was relatively low (28%), the NPV was quite high (94.5%), suggesting that CRP may be useful in ruling out malignancy. Regarding albumin level, a cut-off of 40.75 g/L yielded 68% sensitivity and 72% specificity, with an AUC value of 0.75 (*p* < 0.001). A low albumin level was found to be associated with malignancy and stood out especially due to its high NPV (95%). Although hemoglobin, CRP, and albumin may support the exclusion of malignancy due to their relatively high NPV, they should not be regarded as robust standalone tools. The relatively high NPV observed in our study is largely attributable to the low prevalence of malignancy (8–11%) within the cohort. In higher-risk populations, NPVs would be expected to decline substantially, limiting their generalizability. Therefore, these laboratory parameters may have value primarily in supporting clinical judgment in low-prevalence settings rather than serving as universal exclusion criteria. Based on these findings, it is clear that basic blood tests like hemoglobin, CRP, and albumin can help screen for cancer in patients with thickened bowel walls seen on scans. However, the low PPVs indicate that these parameters alone are not sufficient for diagnosis, and endoscopic and histopathological confirmation remains essential. The number of patients was also insufficient to reliably determine cut-off values, which limits the generalizability of these findings to other centers or populations. Therefore, we believe that these parameters should be tested in larger populations.

There are a few studies on this subject in the literature. Baş et al. retrospectively evaluated 132 patients who had colon wall thickening on CT and underwent colonoscopy. In the study by Baş et al., 132 patients who had thickening of the colon wall on CT and had a colonoscopy were looked at. During the colonoscopies, cancer was found in 38 patients (28.8%), Crohn’s disease in 2 patients (1.5%), diverticulitis in 18 patients (13.6%), and colorectal polyps in 30 patients (22.7%). All patients with colorectal cancer were over the age of 60, and a statistically significant link was found. The mean hemoglobin level in patients with normal colonoscopies was 12.8 g/dL, whereas it was 9.5 g/dL in patients with malignancy. In multivariate analysis, hemoglobin and age were the only variables found to be significant in predicting abnormal findings on endoscopy. The authors concluded that in patients over the age of 50 and with hemoglobin levels below 10 g/dL, the detection of colon wall thickening on CT may suggest malignancy [9]. In the study by Akbaş et al., patients who had endoscopic exams because a CT scan showed bowel wall thickening were split into two groups: one group had normal or non-cancerous results, and the other group had cancerous results. The study investigated whether there were differences between the two groups in terms of hemoglobin, age, albumin, neutrophil-lymphocyte ratio (NLR), platelet-lymphocyte ratio, and mean platelet volume (MPV) values. The results of univariate analysis and multivariate logistic regression identified hemoglobin, NLR, and MPV values as independent predictors in detecting colon cancer [21]. These studies also support the usability of laboratory data for the differential diagnosis of patients with increased wall thickness in radiology.

Our study has several limitations. Firstly, there is no universally accepted guideline establishing a single cut-off value for GI tract wall thickness. The absence of standardized criteria may introduce variability and limit the generalizability of our findings. Secondly, the retrospective and observational design restricts causal inference, and there is a risk of missing or heterogeneous clinical data. Since only patients who underwent endoscopic evaluation were included, the outcomes of those without endoscopy remain unknown, introducing a potential selection bias.

Furthermore, different imaging modalities (CT, MRI, and USG) were included. As these techniques differ in their sensitivity and specificity for detecting wall thickening, their combined use may introduce heterogeneity and diagnostic bias. However, because MRI and USG constituted only a very small proportion of the sample, a reliable subgroup analysis was not feasible. Another limitation is that only ROC curves and basic comparative tests were applied, without multivariate adjustment due to the limited number of malignant cases. As a result, potential confounders could not be controlled, and the findings should be regarded as exploratory. Furthermore, the small number of malignant cases in both upper and lower GI tract subgroups limited the statistical power of ROC analyses and increased the risk of Type I error, and therefore these subgroup results should be interpreted with particular caution. Likewise, no formal correction for multiple comparisons (e.g., Bonferroni) was performed; given that only three laboratory parameters were analyzed and the sample size was limited, unadjusted *p*-values were reported to avoid further loss of statistical power. These statistical constraints mean that our results should be interpreted with caution.

In addition, the sample size was relatively limited for advanced statistical methods such as ROC analysis, and the derived cut-off values require validation in larger cohorts and at different centers. As the study was conducted in only two centers, the findings may not be fully generalizable to other hospitals or healthcare systems, including those in other countries. Operator-dependent variability is also possible, as endoscopic procedures were performed by different physicians, and although standard biopsy protocols were applied, sampling bias cannot be entirely excluded.

Despite all these limitations, our study also has several important strengths. The direct comparison of radiological and endoscopic findings in both upper and lower GI tract segments enhances the originality of the study. Moreover, correlating laboratory parameters with endoscopic diagnoses can be considered a practical approach that may provide benefits to clinicians and contribute to the existing literature.

While not directly investigated in this study, recent advances in artificial intelligence and Internet of Things technologies have shown promise in abdominal radiological imaging, particularly in enhancing diagnostic accuracy and workflow efficiency. These emerging tools may, in the future, contribute to better characterization of GI tract wall thickening and support clinical decision-making. However, their role remains speculative at present, and further dedicated studies are needed before they can be integrated into routine practice.

## 5. Conclusions

GI tract wall thickening detected by abdominal imaging should be considered for endoscopic evaluation, especially when accompanied by clinical and laboratory findings. Disregarding radiological findings may lead to delays in the diagnosis of clinically critical conditions, such as malignancy. Endoscopy remains an important method not only for diagnosis but also for appropriate treatment planning in these patients. Although hemoglobin, CRP, and albumin may support the exclusion of malignancy due to their relatively high NPV, they should not be regarded as adequate standalone screening tools. Large-sample, prospective, multicenter studies including appropriate control groups with normal gastric and intestinal wall morphology are needed to more strongly demonstrate the clinical significance of GI tract wall thickening detected via radiology. These studies will be crucial for determining specific threshold values for different segments, creating risk-based predictive methods, and establishing scoring systems to guide endoscopic evaluation.

## Figures and Tables

**Table 1:** Baseline Characteristics of the Patients.

Characteristics	*n* (%) or Mean (Min–Max)
Age median years (min-max)	59 (18–86)
Sex *n* (%)	209 * (100.0)
Female	115 (55.0)
Male	94 (45.0)
BMI	
<18.5	9 (4.3)
18.5–24.9	70 (33.5)
25–29.9	94 (45.0)
>30	36 (17.2)
ASA physical status	
ASA I	40 (19.1)
ASA II	115 (55.0)
ASA III	54 (25.8)
Comorbid diseases *n* (%)	146 (69.9)
Diabetes mellitus	43 (20.6)
Hypertension	84 (40.2)
Hyperlipidemia	18 (8.6)
Cardiovascular disease	36 (17.2)
Respiratory disease	32 (15.3)
Neuropsychiatric disorder	15 (7.2)
Extra-GI cancer	17 (8.1)
Other (Rheumatologic, urinary system, ...)	38 (18.2)
Radiological imaging modality in which wall thickening was detected	234 * (100.0)
Upper GI tract	142 (60.7)
CT	74 (31.6)
USG	67 (28.6)
MRI	1 (0.4)
Lower GI tract	92 (39.3)
CT	70 (29.9)
USG	20 (8.5)
MRI	2 (0.9)
Hemoglobin (g/dL) (min-max)	13.2 (5.9–17.6)
CRP (mg/L) (min-max)	4 (0.2–245)
Albumin (g/L)	42.1 (14.7–52.0)
WBC (×10^9^/L)	7.5 (3.2–15.8)
Radiological imaging—esophagogastroduodenoscopy interval	24.5 (1–88)
Radiological imaging—ileocolonoscopy interval	32.5 (1–87)

ASA: American Society of Anesthesiologists, BMI: Body Mass Index, CRP: C-Reactive Protein, CT: Computed tomography, GI: Gastrointestinal, MRI: magnetic resonance imaging, USG: Ultrasonography, WBC: White Blood Cell * The number of wall thickening sites (*n* = 234) exceeds the number of patients (*n* = 209) because some patients had more than one GI segment affected.

**Table 2:** Findings of patients who underwent esophagogastroduodenoscopy following radiological imaging.

Number of Patients	142
Endoscopic finding	*n* (%)
Normal	4 (2.8)
Mass in the esophagus	-
Non-reflux esophagitis	-
Reflux esophagitis	26 (18.3)
Hiatal hernia	8 (5.6)
Malignancy	12 (8.5)
Cardia	3 (2.1)
Fundus	-
Corpus	5 (3.5)
Angulus	1 (0.7)
Antrum	3 (2.1)
Polyp	9 (6.3)
Gastritis/erosion	89 (62.7)
Peptic ulcer	9 (6.3)
Gastric	8 (5.6)
Duodenal	1 (0.7)
Duodenitis/celiac disease	4 (2.8)
Mass in the duodenum	2 (1.4)
Other	3 (2.1)

**Table 3:** Findings of ileocolonoscopy performed after radiological imaging.

Number of Patients	92
Endoscopic finding	*n* (%)
Normal	33 (35.9)
Malignancy	10 (10.9)
Cecum	1 (1.1)
Ascending colon	1 (1.1)
Transverse colon	-
Descending colon	-
Sigmoid colon	3 (3.3)
Rectum	5 (5.4)
Anal canal	-
Terminal ileitis	4 (4.3)
Diverticulum	11 (12.0)
Polyp	20 (21.7)
IBD	9 (9.8)
Non-IBD colitis	7 (7.6)
Ulcer	1 (1.1)
Other	3 (3.3)
IBD: Inflammatory bowel disease	

**Table 4:** Findings of Patients Diagnosed with Malignancies and Neoplastic (Adenomatous)/Non-neoplastic Polyps.

Localization and Finding	*n* (%)
Upper GI tract	21/142 * (14.7)
Malignancy	12 (8.5)
Cardia	3 (2.1)
Fundus	-
Corpus	5 (3.5)
Angulus	1 (0.7)
Antrum	3 (2.1)
Polyp	9 (6.3)
Neoplastic	2 (1.4)
Non-neoplastic	7 (4.9)
Lower GI tract	30/92 * (32.6)
Malignancy	10 (10.9)
Cecum	1 (1.1)
Ascending colon	1 (1.1)
Transverse colon	-
Descending colon	-
Sigmoid colon	3 (3.3)
Rectum	5 (5.4)
Anal canal	-
Polyp	20 (21.7)
Neoplastic	14 (15.2)
Non-neoplastic	6 (6.5)

GI: Gastrointestinal * Percentages were calculated based on the number of patients who underwent esophagogastroduodenoscopy (*n* = 142) and ileocolonoscopy (*n* = 92).

**Table 5:** Comparison of Laboratory Findings in Patients With and Without Malignancy Detected by Esophagogastroduodenoscopy and Ileocolonoscopy.

	No Upper GI Tract Malignancy (*n* = 130)	Upper GI Tract Malignancy Present (*n* = 12)	*p* Value
Age median years (min-max)	60 (21–86)	62 (39–81)	0.413 *
Hemoglobin (g/dL) (min-max)	13.1 (8.8–17.5)	10.8 (5.9–13.9)	<0.001 *
CRP (mg/L) (min-max)	4 (0.2–187)	22.5 (1.7–122)	0.015 *
Albumin	42.2 (14.7–52)	37.5 (28–44)	0.005 *
	**No lower GI tract malignancy (*n* = 82)**	**Lower GI tract malignancy present (*n* = 10)**	***p* value**
Age median years (min-max)	56 (18–83)	68.5 (34–77)	0.081 *
Hemoglobin (g/dL) (min-max)	13.6 (9–17.6)	11 (8.8–15.3)	0.033 *
CRP (mg/L) (min-max)	4 (0.4–245)	16.7 (0.4–121)	0.115 *
Albumin	42.2 (18–50)	39 (22–43)	0.015 *

* Mann–Whitney U test. CRP: C-Reactive Protein, GI: Gastrointestinal.

## Data Availability

The original contributions presented in this study are included in the article. Further inquiries can be directed to the corresponding author.

## Abbreviations

The following abbreviations are used in this manuscript:

AUC	Area Under the Curve
BMI	Body Mass Index
CI	Confidence Interval
CRP	C-Reactive Protein
CT	Computed Tomography
GI	Gastrointestinal
HGB	Hemoglobin
IBD	Inflammatory Bowel Disease
MRI	Magnetic Resonance Imaging
NPV	Negative Predictive Value
PPV	Positive Predictive Value
ROC	Receiver Operating Characteristic
USG	Ultrasonography
